# Discovery of novel and potent celastrol derivatives as PRDX1 inhibitors for cancer therapy through structure-based virtual screening

**DOI:** 10.3389/fphar.2025.1625604

**Published:** 2025-07-23

**Authors:** Lixia Guan, Yifei Geng, Yuting Wang, Miao-Miao Niu, Kun Shi

**Affiliations:** ^1^Department of Pharmaceutical Analysis, China Pharmaceutical University, Nanjing, China; ^2^Department of Orthopedics, Xuzhou Central Hospital, Xuzhou, Jiangsu, China

**Keywords:** Prdx1, ROS, base-structure virtual screening, molecular docking, cancer cells

## Abstract

**Background:**

Peroxiredoxin 1 (PRDX1) is an antioxidant enzyme overexpressed in several cancers that protects tumor cells from oxidative damage by scavenging excess reactive oxygen species making it a potential strategy for cancer therapy.

**Methods:**

In this study, a multi-step screening strategy combining molecular docking, enzyme inhibition assay, enzyme kinetic studies, molecular dynamics (MD) simulations, MST assays, MTT assays and *in vivo* toxicity assay was used to discover PRDX1 inhibitors.

**Results:**

Five compounds (CPs 1–5) targeting PRDX1 were identified through molecular docking screening. CPs 1-5 showed significant PRDX1 inhibition at the nanomolar level. Among them, CP1 exhibited the most potent inhibitory activity (IC_50_ = 0.08 ± 0.01 nM) and high selectivity against PRDX1. The kinetic study showed that CP1 acted as noncompetitive PRDX1 inhibitor. MD simulations confirmed the stability of the CP1-PRDX1 complex. MST assays revealed that CP1 displayed a significant binding affinity for PRDX1 (*K*_d_ = 0.06 ± 0.001 nM). Importantly, CP1 exhibited significant antiproliferative effects on A549, HepG2 and MCF-7 tumor cells without toxicity to other normal cells. Meanwhile, CP1 did not exhibit significant hepatotoxicity or renal toxicity in mice.

**Conclusion:**

The results suggest that CP1 is a promising antitumor candidate for cancer therapy and merits further investigation.

## 1 Introduction

Cancer, a complex and heterogeneous disease, is a major global public health problem (Siegel et al., 2023; Chandrashekar et al., 2022). The escalating global burden of cancer has emerged as a major factor in premature death and reduced life expectancy in many countries (Cao et al., 2021; Brustugun et al., 2014). Cancer has a multifaceted mechanism of initiation and progression characterized by excessive proliferation, angiogenesis, and resistance to apoptosis, migration and immune evasion, making cancer treatment a complex process (de Sa et al., 2017). Currently, traditional cancer therapies, including surgery, chemotherapy and radiotherapy, lack specificity and often result in the destruction of normal cells, leading to severe side effects and toxicity (Debela et al., 2021; Conibear et al., 2020). Therefore, the discovery of novel anticancer agents remains an urgent need.

ROS are a class of highly reactive oxygen-containing free radicals that play critical roles in the regulation of physiological homeostasis and pathological processes (Sahoo et al., 2022; Perillo et al., 2020). Dysregulation of redox homeostasis is a feature of many malignant tumors, manifested by persistently elevated intracellular ROS levels (Lin et al., 2018; Saikolappan et al., 2019). Excessive ROS accumulation induces oxidative damage to DNA, proteins, and lipids, ultimately triggering cellular apoptosis or necrosis (Redza-Dutordoir and Averill-Bates, 2016; Juan et al., 2021). Tumor cells rely on antioxidant enzymes to scavenge excess ROS to maintain survival, suggesting that inhibition of these antioxidant enzymes may represent a promising strategy for tumor therapy (Pelicano et al., 2004). Studies have shown that inhibiting antioxidant enzymes can induce more severe ROS-mediated damage in cancer cells than in normal cells, and some drugs targeting antioxidant enzymes have now entered clinical trials (Pelicano et al., 2004).

**FIGURE 1:**
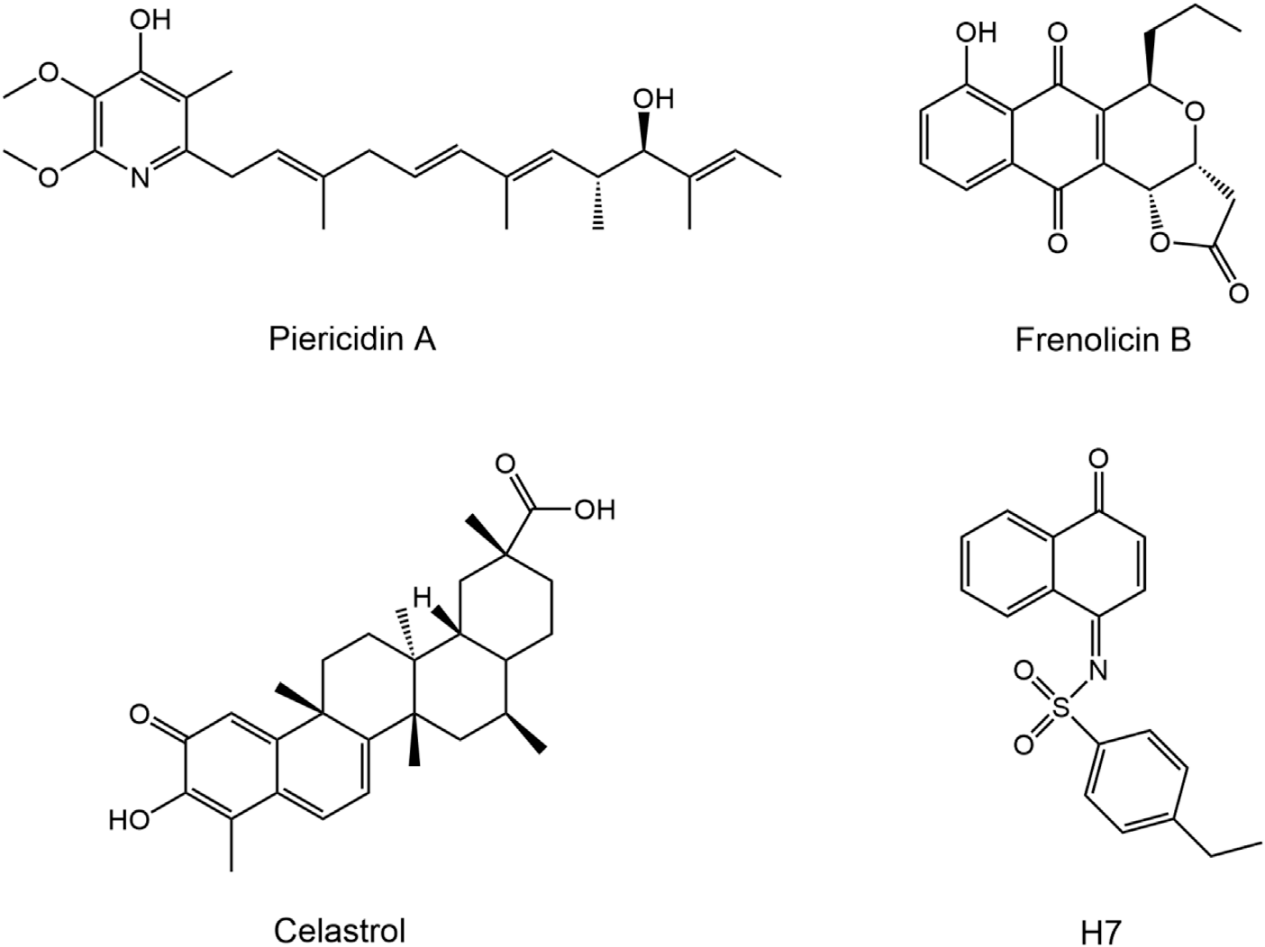
Reported PRDX1 inhibitors.

Peroxiredoxins (PRDXs), a family of antioxidant proteins, are essential regulators of diverse biological processes by modulating the metabolism of hydrogen peroxide (H_2_O_2_) and other ROS (Bajor et al., 2018; Kim and Jang, 2019). PRDXs are classified into six subtypes (PRDX 1–6) based on the conserved cysteine residues: i) PRDX 1–4 (typical 2-Cys); ii) PRDX5 (atypical 2-Cys); and iii) PRDX6 (1-Cys) (Ding et al., 2017; Sun et al., 2015). PRDX1 is a multifunctional antioxidant protein that regulates critical cellular processes, including proliferation, differentiation, apoptosis, migration, and angiogenesis (Hampton et al., 2018; Cai et al., 2015). As a key intracellular hub, PRDX1 modulates multiple ROS-dependent signaling cascades, thereby maintaining a dynamic balance between cell survival and apoptotic pathways (Ding et al., 2017; Ishii et al., 2012). Aberrant overexpression of PRDX1 in malignancies promotes oncogenic phenotypes manifested by enhanced invasive potential through oxidative stress damage, facilitation of malignant transformation and acquisition of chemo-/radiotherapy resistance (Bae et al., 2007; Li et al., 2024). Elevated PRDX1 expression has been observed in several human tumors, including lung, breast, liver, colon and other cancers (Lu et al., 2020). Studies have shown that targeting PRDX1 can inhibit the proliferative capacity of breast cancer cells (Bajor et al., 2018). Thus, PRDX1 is a promising therapeutic target for the development of novel anticancer drugs. To date, some small molecule inhibitors targeting PRDX1 have been identified, including piericidin A, frenolicin B, H7 and celastrol (Figure 1) (Zhou X. F. et al., 2019; Ye et al., 2019; Wei et al., 2016; Xu et al., 2023). Celastrol, an extensively studied natural product with antitumor activity, exhibits significant cytotoxic effects on colorectal cancer cells by targeting PRDX1 (Shi et al., 2020; Moreira et al., 2019). However, further clinical application is severely limited by systemic toxicities, such as cytotoxicity, hepatotoxicity and renal toxicity, primarily due to off-target effects (Sun et al., 2024; Wang et al., 2024). Thus, the development of PRDX1 inhibitors with high efficacy and low toxicity remains a major challenge.

Structure-based virtual screening is the systematic computational analysis to identify potential drug candidates against specific therapeutic targets in a cost-effective and efficient manner (Ma et al., 2011; Zhang et al., 2022). Molecular docking allows the prediction of ligand-target binding conformations and binding free energies, facilitating the efficient identification of potential leads with optimal binding affinity (Hosseini et al., 2021; Forli et al., 2016). In previous studies, we have successfully discovered several novel inhibitors by base-structure virtual screening (Zhou et al., 2022; Yang et al., 2020; Zheng et al., 2021; Zheng et al., 2023; Zhou et al., 2021). Here, an integrated virtual screening platform was established by integrating molecular docking, MD simulations and biological evaluation, which enabled the efficient and accurate discovery of potential lead compounds (CPs 1–5). Enzyme inhibition assays revealed that these compounds exhibited significant inhibitory activity against PRDX1. CP1 exhibited the strongest inhibitory activity and remarkable selectivity toward PRDX1. Meanwhile, CP1 functioned as a noncompetitive inhibitor of PRDX1. MST assays revealed that CP1 had a significant binding affinity for PRDX1. Importantly, CP1 showed potent antiproliferative effects against tumor cells without observable cytotoxicity, indicating its potential as a therapeutic candidate.

## 2 Materials and methods

### 2.1 Cell culture and materials

The cells were purchased from the American Type Culture Collection (ATCC) (Manassas, VA, United States). Cells were cultured in RPMI-1640 medium supplemented with 10% FBS, 50 units/mL penicillin, and 50 μg/mL streptomycin, and maintained at 37°C in a 5% CO_2_ humidified culture environment. CPs 1-5 were purchased from WuXi AppTec. Human PRDX1 proteins were purchased from Abcam (Cambridge, MA, United States).

### 2.2 Docking-based virtual screening

The crystal structure of PRDX1 protein (PDB ID: 4XCS) was obtained from the PDB database. The structure was imported into the Molecular Operating Environment (MOE) software and protein was preprocessed in the Amber10:EHT force field using the QuickPrep tool, including energy minimization of the protein, addition of hydrogen atoms, and complementation of missing residues. Meanwhile, the 2D structures of compounds from a 35,000-molecule database were converted into 3D conformations using MOE’s Conformation Import tool under the MMFF94 force field. Subsequently, molecular docking was performed using Dock tool of MOE. Docking screening was performed from the database of 35,000 compounds based on the binding positions of the original ligands of the proteins. Molecular docking was performed using the triangle matching algorithm of the Dock tool and the dG docking score to calculate the binding free energy. In general, the lower the binding free energy, the better the binding affinity. Molecular interactions between protein and ligand were analyzed using the Ligand Interactions tool and maps of protein binding pockets were generated using the Surface and Maps tool.

### 2.3 PRDX1 inhibition assay

First, 120 μM buffer (20 mM HEPES, 5 mM EDTA, 5 μM cofactor A, 2 μM cofactor B, and 300 μM NADPH) was added to a 96-well plate. The test compounds at the designated concentration (0.02–2 nM, with 1.5-fold dilution) were incubated with PRDX1 at 37°C for 25 min, and then the co-incubated mixture was added to each well to a final concentration of 200 nM PRDX1. Finally, 200 μM H_2_O_2_ was added to each well to initiate the enzyme reaction cycle. The oxidation of NADPH was determined by the absorption value at 340 nm. Read plate values for 90 s for one cycle and repeat for 20 cycles. The positive control (celastrol, concentrations 0.06–64 μM) was tested in parallel under identical experimental conditions. Three independent replicate experiments were performed.

### 2.4 Kinase selectivity assay

The selectivity of CP1 for PRDX1 was evaluated through kinase selectivity assays against other PRDX family members and a panel of kinases. The experiments were conducted by ICE Biosciences (Beijing, China).

### 2.5 Kinetics studies of PRDX1 inhibition

The PRDX1 protein (0.5 nM) was incubated with varying concentrations of the CP1 (0, 0.6, 1.5, and 3.8 nM) at room temperature for 1 h. Subsequently, the Trx/TrxR/NADPH mixture was added and the enzymatic reaction was initiated by the addition of different concentrations of H_2_O_2_ (5–1,000 μM). The absorbance changes at 340 nm were monitored in real-time using a microplate reader. Kinetic parameters, including the Michaelis constant (K_m_), maximum reaction rate (V_max_), and α value, were determined by fitting the data to the Michaelis-Menten equation.

### 2.6 MD simulation

The MD simulations were performed using GROMACS with AMBER99SB-ILDN force field. The structure of PRDX1 was obtained from the PDB (PDB ID: 4XCS). PRDX1 was imported into GROMACS 2021.5 and topology files were produced. Topology files for small molecule inhibitors were generated using Acpype. The protein-ligand complex system was placed in the center of a cubic box. The complex was solvated with the SPC/E water model and neutralized by the addition of appropriate Na^+^ and Cl^−^ ions. Then, the neutralized system was subjected to a 5000-step energy minimization using the steepest descent method. Furthermore, a NVT simulation was carried out with a V-rescale thermostat to maintain the temperature of the complex system at 300 K, and a NPT simulation was conducted with a Parinello-Rahman barostat to maintain the pressure of the complex system at 1 atm. Finally, 50 ns MD simulations were performed and the data were analyzed using Prism 8.0 software.

### 2.7 MST assay

The MST assays were carried out in a buffer composed of 50 mM Tris, 200 mM NaCl, and 0.1% (v/v) TWEEN20. PRDX1 was tagged with the MO-L011 protein labeling kit (RED-NHS second Generation). The diluted compounds were combined with the tagged PRDX1 protein and allowed to stand at room temperature for 5 minutes. Subsequently, the reaction mixture was loaded into capillaries, and measurements were taken using a Monolith NT.115 instrument.

### 2.8 MTT assay

Cells were seeded in 96-well plates at 5 ×10^4^ cells/well and cultured overnight at 37°C under 5% CO_2_. After medium removal, cells were treated with serially diluted compounds (0.04–163.84 μM) or vehicle control (0.1% DMSO) and incubated for 72 h. Subsequently, 20 μL of MTT solution (5 mg/mL) was added to each well, and incubation was continued for 4 h. Add 150 μL of DMSO and shake for 10 min to fully dissolve the crystal. Finally, the absorbance was measured at 570 nm using a microplate reader. Cell viability was calculated as (OD_compound_ - OD_blank_)/(OD_vehicle_ - OD_blank_) × 100%. The half inhibitory concentration (IC_50_) was calculated from the dose-response curve using Prism 8.0 software. Three independent replicate experiments were performed.

### 2.9 *In vivo* toxicity assay

To evaluate the *in vivo* toxicity of CP1, C57BL/6 mice were divided into three groups and received a single intraperitoneal injection: vehicle, CP1 (5 mg/kg), and CP1 (10 mg/kg). Blood samples were collected after 24 h, and hepatic and renal function indicators including alanine aminotransferase (ALT), aspartate aminotransferase (AST), blood urea nitrogen (BUN), and creatinine (CRE) were detected using a fully automated biochemical analyzer.

### 2.10 Statistical analysis

All experiments were performed in three times and results are presented as mean ± standard deviation. Statistical analyses were conducted using ANOVA (Dunnett’s test), and p values less than 0.05 were considered significant.

## 3 Results

### 3.1 Virtual screening

**FIGURE 2:**
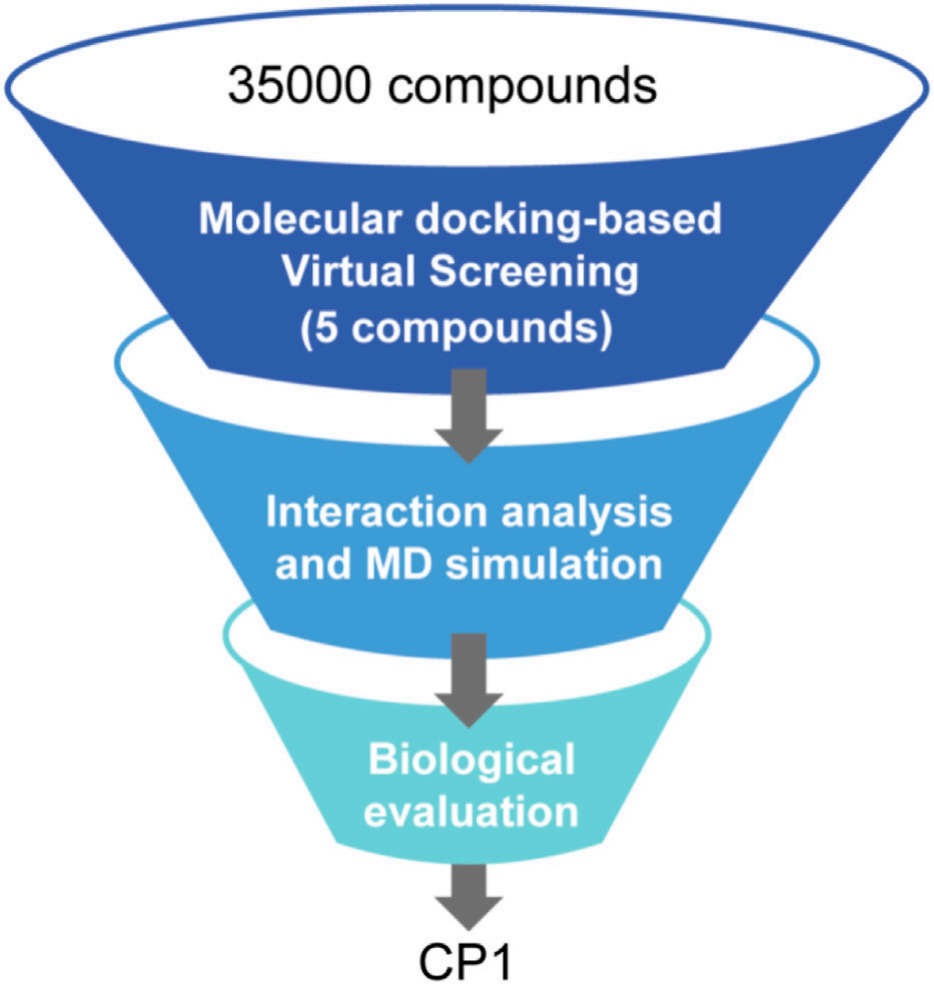
The workflow of multi-step virtual screening.

**FIGURE 3:**
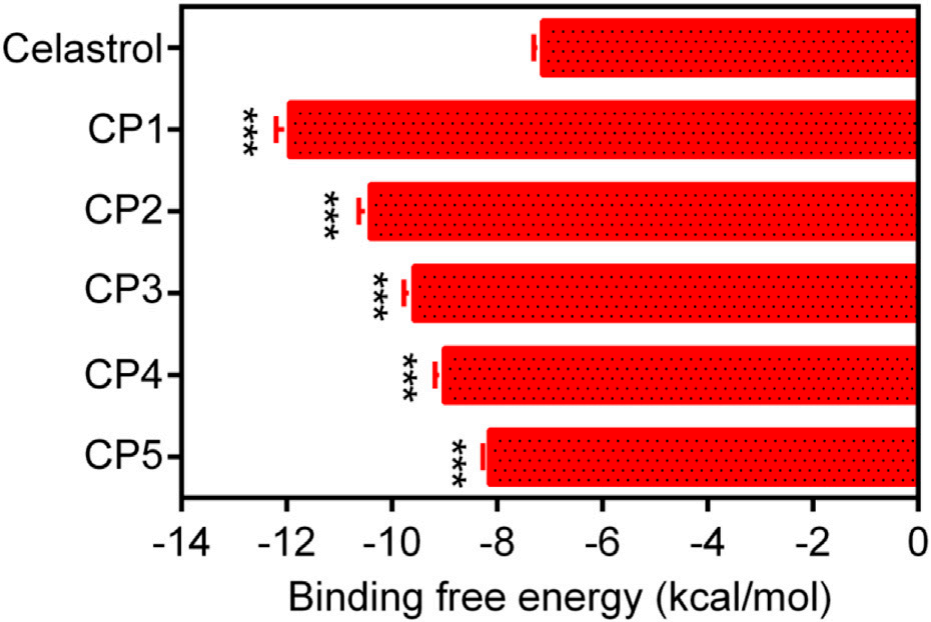
The binding free energy (kcal/mol) of CPs 1–5. Celastrol served as the positive controls. Data are expressed as mean ± SD, n = 3. For CPs 1-5 groups, ***P < 0.001 means a significant difference *versus* celastrol.

**FIGURE 4:**
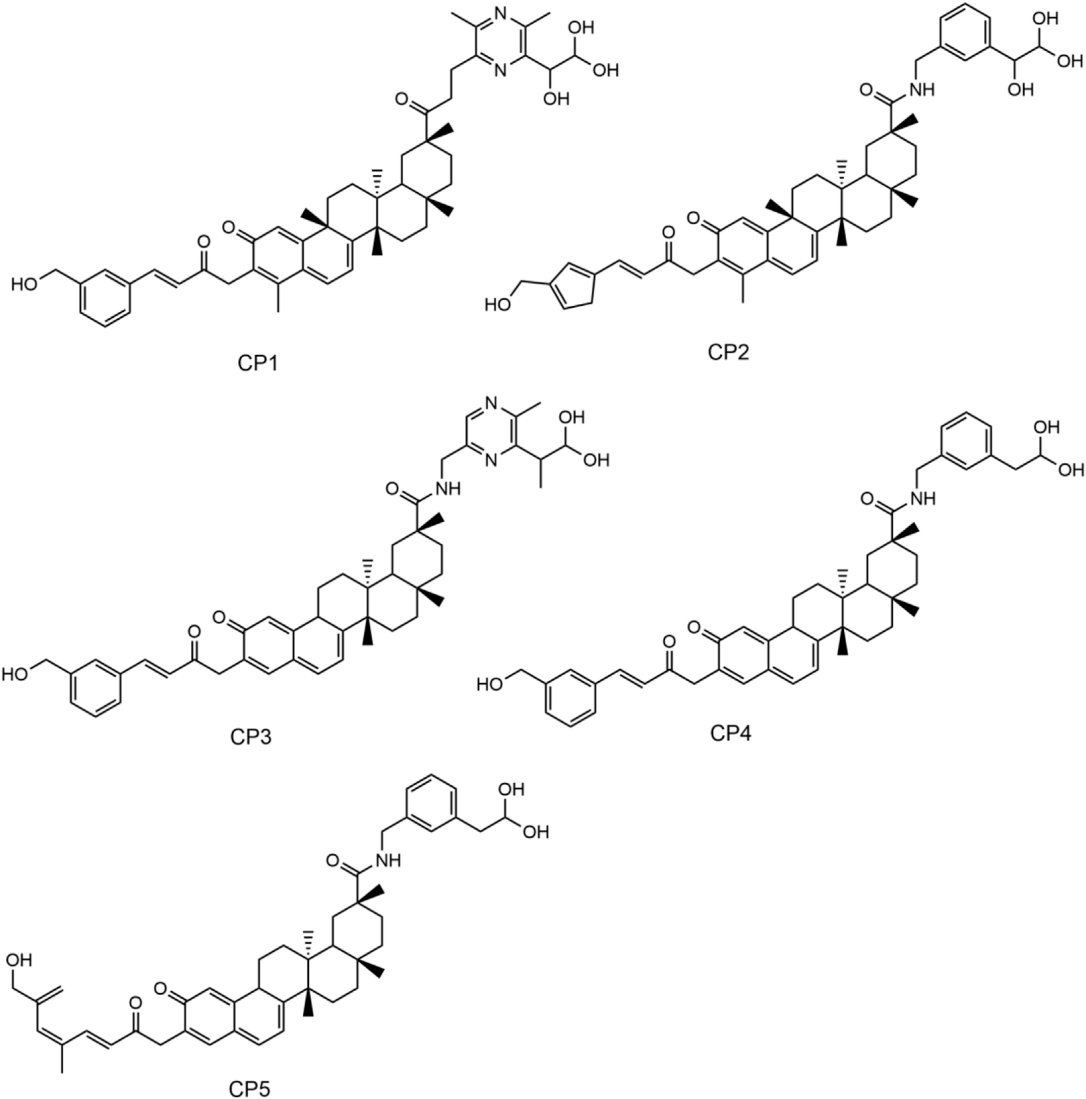
The chemical structures of CPs 1-5.

**FIGURE 5:**
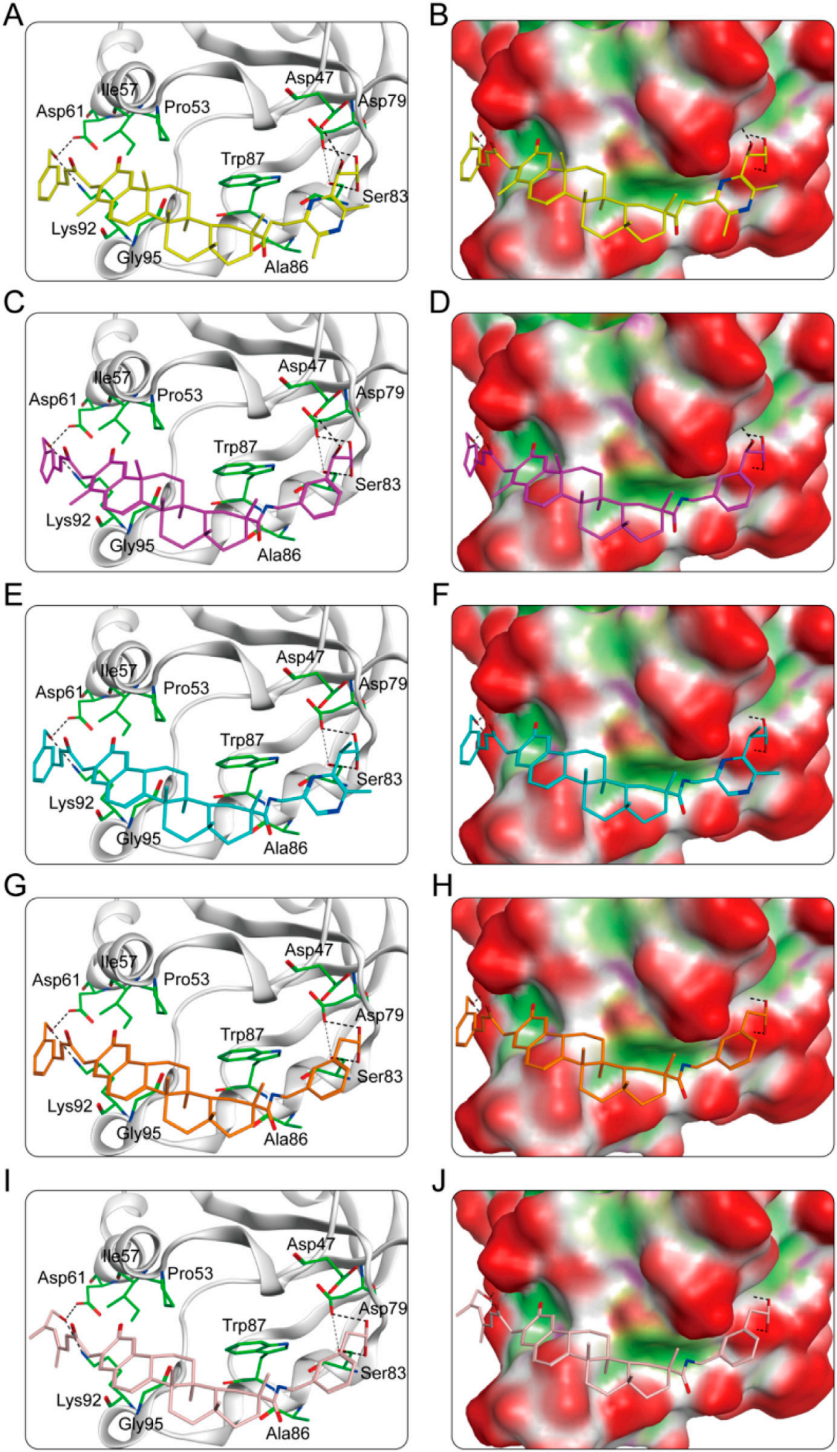
**(A)** The interaction patterns of CP1 within the PRDX1 binding site; **(B)** The binding pocket of CP1 within the PRDX1 binding site; **(C)** The interaction patterns of CP2 within the PRDX1 binding site; **(D)** The binding pocket of CP2 within the PRDX1 binding site; **(E)** The interaction patterns of CP3 within the PRDX1 binding site; **(F)** The binding pocket of CP3 within the PRDX1 binding site; **(G)** The interaction patterns of CP4 within the PRDX1 binding site; **(H)** The binding pocket of CP4 within the PRDX1 binding site; **(I)** The interaction patterns of CP5 within the PRDX1 binding site; **(J)** The binding pocket of CP5 within the PRDX1 binding site. The residues are shown as green bars, while hydrogen bonds are shown as black dashed lines.

In this study, a multilevel screening strategy integrating structure-based virtual screening and experimental validation was used to identify novel inhibitors targeting PRDX1. As shown in Figure 2, the comprehensive screening workflow consisted of three main phases: (1) initial molecular docking screening to assess the binding affinity between compounds and the PRDX1; (2) MD simulations to assess the binding stability of potential inhibitors with PRDX1; (3) biological validation. For the structure-based virtual screening process, a 2D structural database of 35,000 compounds (Zhou Y. et al., 2019) was converted into 3D conformations. Next, these compounds were precisely docked into the active pocket of the PRDX1 protein by molecular docking, and the binding affinity was quantitatively characterized by the binding free energy calculated using the London dG scoring function. A greater negative binding free energy (a lower docking score) indicates a higher binding affinity. Meanwhile, molecular docking analysis was employed to evaluate the binding modes between the compounds and the PRDX1 protein, leading to the exclusion of compounds that lacked direct interactions with the key residues of PRDX1. Finally, the five lowest scoring compounds (CPs 1–5) were screened as candidates with significantly lower binding free energies than the positive control celastrol (Figure 3). Structural analysis revealed that the groups at the ends of CPs 1-5 formed four stable hydrogen bonds with the key residues of PRDX1, suggesting that these compounds have superior binding affinity (Figure 5). Notably, CP1 had the lowest binding free energy, suggesting the strongest binding affinity to PRDX1 of all the compounds screened. The structures of CPs 1-5 are shown in Figure 4.

### 3.2 Interaction analysis

The key molecular interactions of ligand-target binding and their structure-activity relationship (SAR) were revealed using a molecular docking software system. The molecular docking patterns and interaction characteristics of CPs 1-5 with PRDX1 are comprehensively analyzed in Figure 5. The SAR analysis is presented in Table 1. CPs 1-5 formed stable hydrophobic interactions with key hydrophobic residues (Pro53, Ile57, Ala86, Trp87 and Gly95) in the active pocket of PRDX1. Meanwhile, CP1 and CP2 exhibited superior hydrogen bonding properties as evidenced by the formation of five hydrogen bonds with residues (Asp47, Asp61, Asp79, Ser83 and Lys92) of PRDX1 compared to the other compounds which formed four hydrogen bonds. Molecular surface analysis further confirms a good spatial match between the PRDX1 active site pockets and the structure of CPs 1-5, suggesting strong structural complementarity. In particular, the additional hydrogen bonds formed by the terminal groups of CP1 and CP2 with Asp47 in the PRDX1 active site contributed to their enhanced binding stability within the PRDX1 active pocket, resulting in a more stable binding conformation. In summary, structural analyses showed that CPs 1-5 exhibited significant molecular interactions with key residues in the PRDX1 active site, with CP1 and CP2 exhibiting particularly strong binding properties.

**TABLE 1:** The structure-activity relationship analysis of CPs 1-5 with PRDX1.

Name	Hydrogen bonding interactions		Hydrophobic interactions
	Amino acid involved	H-bond numbers	
CP1	Asp47	1	Pro53, Ile57, Ala86, Trp87, Gly95
	Asp61	1	
	Asp79	1	
	Ser83	1	
	Lys92	1	
CP2	Asp47	1	Pro53, Ile57, Ala86, Trp87, Gly95
	Asp61	1	
	Asp79	1	
	Ser83	1	
	Lys92	1	
CP3	Asp61	1	Pro53, Ile57, Ala86, Trp87, Gly95
	Asp79	1	
	Ser83	1	
	Lys92	1	
CP4	Asp61	1	Pro53, Ile57, Ala86, Trp87, Gly95
	Asp79	1	
	Ser83	1	
	Lys92	1	
CP5	Asp61	1	Pro53, Ile57, Ala86, Trp87, Gly95
	Asp79	1	
	Ser83	1	
	Lys92	1	

### 3.3 *In vitro* PRDX1 inhibitory activity

To systematically evaluate the inhibitory effects of CPs 1-5 on PRDX1, we performed quantitative analysis using enzyme inhibition assays. As shown in Table 2, CPs 1-5 showed significant inhibitory activity against PRDX1, with IC_50_ values ranging from 0.08 to 0.36 nM. This indicates a nanomolar potency advantage over the positive control celastrol (IC_50_ = 0.51 ± 0.04 μM). Notably, CP1 showed the strongest inhibitory potency (IC_50_ = 0.08 ± 0.01 nM) among the tested compounds.

**TABLE 2:** The inhibitory activities of CPs 1-5 against PRDX1, celastrol served as the positive controls.

Compounds	PRDX1 (IC_50_, nM)
CP1	0.08 ± 0.01
CP2	0.17 ± 0.02
CP3	0.25 ± 0.01
CP4	0.31 ± 0.03
CP5	0.36 ± 0.02
Celastrol	0.51 ± 0.04 μM

The data are presented as the mean ± SD, n = 3.

Furthermore, kinase selectivity assay was conducted against other PRDX family members and a panel of kinases to further evaluate the selective inhibition of PRDX1 by CP1. As shown in Supplementary Table S1, CP1 exhibited minimal inhibitory activity against these kinases (IC_50_ >30 μM). These results indicate that CP1 selectively inhibits PRDX1.

### 3.4 Noncompetitive inhibition of PRDX1 by CP1

The kinetic parameters (V_max_, K_m_ and α value) were determined to clarify the inhibition mode of CP1. As shown in Supplementary Table S2, the V_max_ values decreased gradually while the K_m_ values increased with rising inhibitor concentrations. The α value for CP1 was greater than 1.0, indicating that its affinity for free PRDX1 protein was higher than for the PRDX1/H_2_O_2_ complex. These results suggest that CP1 is a noncompetitive inhibitor of PRDX1.

### 3.5 MD simulation

**FIGURE 6:**
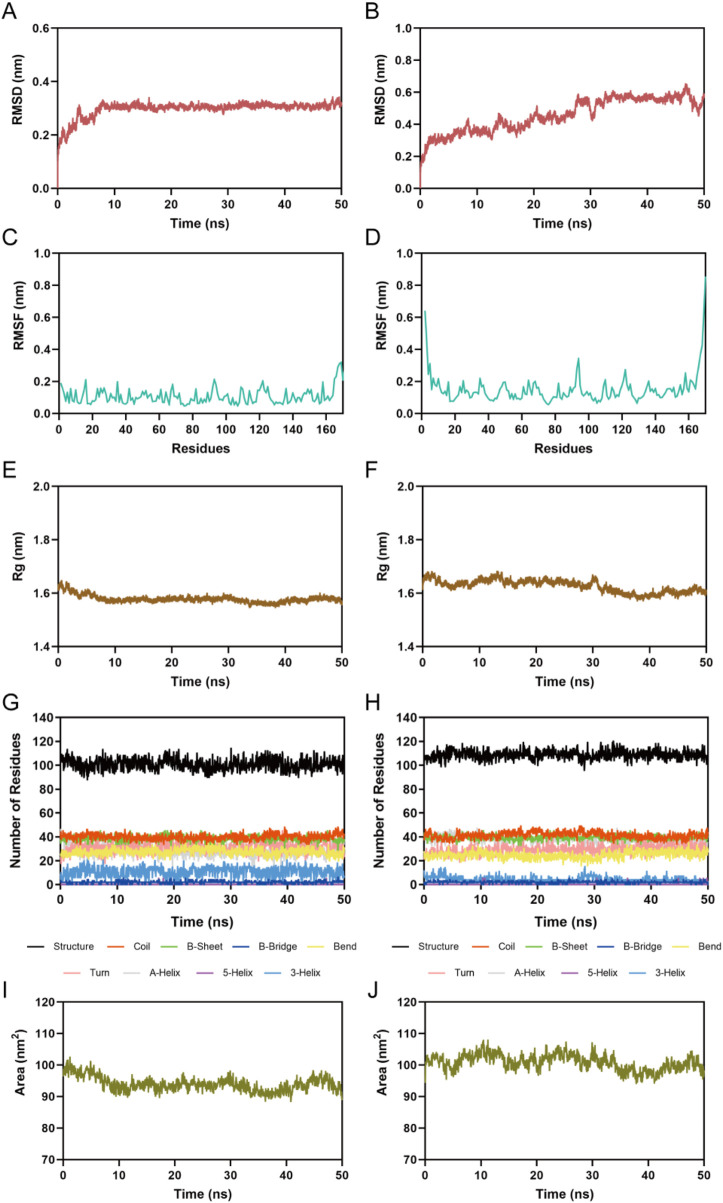
MD simulation of PRDX1 in complex with CP1 and CP2. **(A)** RMSD of the CP1-PRDX1 complex; **(B)** RMSD of the CP2-PRDX1 complex; **(C)** RMSF of PRDX1 residues in the CP1-PRDX1 complex; **(D)** RMSF of PRDX1 residues in the CP2-PRDX1 complex; **(E)** Rg of PRDX1 in the CP1-PRDX1 complex; **(F)** Rg of PRDX1 in the CP2-PRDX1 complex; **(G)** The secondary structures of PRDX1 in the CP1-PRDX1 complex; **(H)** The secondary structures of PRDX1 in the CP2-PRDX1 complex; **(I)** SASA of PRDX1 in the CP1-PRDX1 complex; **(J)** SASA of PRDX1 in the CP2-PRDX1 complex.

CP1 and CP2 showed significant inhibitory activity against PRDX1. To evaluate the binding stability of the CP1-PRDX1 and CP2-PRDX1 complexes, we further conducted 50 ns MD simulations using the GROMACS software. The MD simulation results showed that the equilibrium temperatures of the CP1-PRDX1 and CP2-PRDX1 systems were 300.002 K and 300.006 K, respectively, with pressures of 1.10 bar and 1.57 bar, meeting the predefined thermodynamic conditions. The dynamic behavior and stability of the protein-ligand complexes were assessed using key MD parameters: root mean square deviation (RMSD), root mean square fluctuation (RMSF), radius of gyration (Rg), protein secondary structure, and solvent accessible surface area (SASA). The structural stability of the complexes is indicated by the deviation in time-scale simulations of these key parameters. RMSD is a metric that calculates the average deviation of atomic positions in the molecular structure from the initial conformation, and a levelling off of the RMSD indicates convergence of the system. As shown in Figures 6A,B, the RMSD values of the CP1-PRDX1 complex initially showed a slight increase and stabilized around 0.3 nm after 10 ns, whereas the RMSD of the CP2-PRDX1 complex demonstrated a continuous gradual upward trend. These results indicate that CP1 forms a stable binding complex with PRDX1. RMSF measures the fluctuation amplitude of each residue in a protein structure relative to its average position, whereby lower values indicate reduced residue movement and enhanced structural stability. In Figures 6C,D, the RMSF values of the key residues Asp47, Pro53, Ile57, Asp61, Asp79, Ser83, Ala86, Trp87, Lys92, and Gly95 in the binding site of PRDX1 were less than 0.20 nm, suggesting stable interactions between the key amino acids of PRDX1 with CP1 and CP2. Rg is an indicator of the structural compactness of a protein, with small fluctuations in Rg indicating structural stability. As shown in Figures 6E,F, PRDX1 bound to CP1 exhibited greater structural compactness with Rg maintained at a stable level of 1.58 nm, which was significantly more stable than PRDX1 bound with CP2. This suggests that CP1 is more effective in maintaining the structural integrity of PRDX1. As shown in Figures 6G,H, the secondary structure of PRDX1 remained stable, indicating that binding with CP1 and CP2 did not induce significant conformational changes in the protein. The SASA refers to the surface area of a protein that interacts with solvent molecules. Figures 6I,J showed that the SASA of PRDX1 in the CP1-PRDX1 complex decreased continuously to about 90 nm^2^ within the first 10 ns and maintains stable fluctuations, suggesting that ligand binding induces a decrease in the surface-exposed region of the protein. The SASA of PRDX1 in the CP2-PRDX1 complex is remained around 100 nm^2^. This suggests that CP1 induces a more surface-buried binding conformation of PRDX1 than CP2, which may be related to its stronger interfacial interactions. In summary, CP1 showed a more stable binding with the PRDX1 compared to CP2, maintaining the structural stability of the complex during the simulation period. In addition, a 50 ns MD simulation was performed on the celastrol-PRDX1 complex. As shown in Supplementary Figure S1A, the RMSD value of the complex exhibited a gradual upward trend, indicating that its binding state was relatively unstable. The RMSF values of key residues (Asp47, Pro53, Ile57, Asp61, Asp79, Ser83, Ala86, Trp87, Lys92, and Gly95) in the PRDX1 binding site are all less than 0.20 nm (Supplementary Figure S1B). The Rg of PRDX1 remained stable at 1.58 nm (Supplementary Figure S1C) and there were no significant changes to its secondary structure (Supplementary Figure S1D). The SASA of PRDX1 in the celastrol-PRDX1 complex fluctuated within the range of 90–100 nm^2^ (Supplementary Figure S1E), which was slightly higher than the surface-exposed area of PRDX1 induced by CP1 binding. In conclusion, CP1 binds more stably to PRDX1 than celastrol.

The total binding free energy was calculated using the MM/PBSA method. As shown in Table 3, the total binding free energies of the CP1-PRDX1 and CP2-PRDX1 complexes were −43.73 and −42.22 kcal/mol, respectively. For both compounds, the van der Waals energy, electrostatic energy, and total gas-phase free energy were the major contributors to binding. Additionally, the total binding free energy of the celastrol-PRDX1 complex was −17.12 kcal/mol, suggesting that the binding capacity of celastrol to PRDX1 was lower than that of CP1 to PRDX1 (Supplementary Table S3). Overall, CP1 showed stronger binding affinity to PRDX1 than CP2 and celastrol.

**TABLE 3:** Predicted free energies (kcal/mol) for the binding of CP1 and CP2 to PRDX1.

Energy terms (kcal/mol)	CP1	CP2
van der Waals energy	−37.09 ± 1.71	−47.87 ± 2.55
electrostatic energy	−51.75 ± 1.91	−49.14 ± 5.66
polar solvation energ	50.55 ± 1.89	61.28 ± 4.30
nonpolar solvation energy	−5.44 ± 0.18	−6.50 ± 0.24
total gas-phase free energy	−88.84 ± 2.86	−97.00 ± 6.04
total solvation free energy	45.11 ± 1.78	54.78 ± 4.14
total binding free energy	−43.73 ± 1.73	−42.22 ± 2.92

The data are presented as the mean ± SD.

### 3.6 Binding affinity assay

The binding affinity between CP1 and PRDX1 was determined by MST assay, with celastrol as control. As shown in Supplementary Table S4, CP1 exhibited a significantly higher binding affinity to PRDX1 (*K*_d_ = 0.06 ± 0.001 nM) compared to the control celastrol (*K*_d_ = 0.32 ± 0.02 μM). This suggests that CP1 represents a highly promising PRDX1 inhibitor.

### 3.7 Inhibition of cell growth

The antiproliferative effects of CPs 1-5 on A549 lung cancer cells and BEAS-2B normal lung cells were evaluated using the MTT assay. As shown in Table 4, CPs 1-5 exhibited significant antiproliferative activity against A549 cells, with potency significantly superior to the positive control celastrol. Notably, CP1 showed the strongest antiproliferative activity against A549 cells (IC_50_ = 0.22 ± 0.01 μM), which was approximately 5-fold improvement compared to celastrol (IC_50_ = 1.24 ± 0.08 μM). More importantly, CPs 1-5 showed no significant antiproliferative effects on BEAS-2B normal lung cells (IC_50_ >100 μM), whereas celastrol exhibited significant growth inhibition (IC_50_ = 0.45 ± 0.03 μM).

**TABLE 4:** Antiproliferative effects of CPs 1-5 against A549 and BEAS-2B cells by MTT assay, celastrol served as the positive controls.

Compounds	IC_50_ (μM)	
	A549	BEAS-2B
CP1	0.22 ± 0.01	>100
CP2	0.46 ± 0.03	>100
CP3	0.57 ± 0.05	>100
CP4	0.63 ± 0.04	>100
CP5	0.68 ± 0.06	>100
Celastrol	1.24 ± 0.08	0.45 ± 0.03

The data are presented as the mean ± SD, n = 3.

In addition, MTT assays were conducted on several other tumor cell lines, including HepG2 (hepatocellular carcinoma cells) and MCF-7 (breast adenocarcinoma cells), as well as on human normal cells, including HEK293 (embryonic kidney cells), LO2 (normal liver cells) and HaCaT (immortalised keratinocytes). As shown in Supplementary Table S5, CP1 exhibited significant antiproliferative activity against HepG2 (IC_50_ = 0.25 ± 0.02 μM) and MCF-7 (IC_50_ = 0.27 ± 0.01 μM), with its efficacy being notably superior to that of the control celastrol (IC_50_ = 1.28 ± 0.05 μM for HepG2 and IC_50_ = 1.31 ± 0.06 μM for MCF-7). Meanwhile, CP1 showed almost no cytotoxicity toward these normal human cells (IC_50_ >100 μM), whereas celastrol exerted strong cytotoxicity against these normal cells: HEK293 (IC_50_ = 5.76 ± 0.12 μM), LO2 (IC_50_ = 24.04 ± 0.69 μM), HaCaT (IC_50_ = 17.38 ± 0.54 μM). These results indicate that CP1 has remarkable antiproliferative activity against these tumor cells without toxicity to normal cells compared to celastrol. Based on these results, CP1 has significant therapeutic potential in the treatment of cancer, due to its excellent antiproliferative activity and superior safety profile.

### 3.8 *In vivo* toxicity assay

To evaluate the toxic effects of CP1 in mice, we detected liver and kidney function indicators. As shown in Supplementary Figure S2, there were no significant differences in the levels of the indicators of liver and kidney function (ALT, AST, BUN, and CRE) among the groups. These results suggest that CP1 did not show obvious hepatotoxicity or renal toxicity.

## 4 Discussion

The imbalance of redox homeostasis is an important feature of malignant tumors, and persistently elevated levels of ROS can induce oxidative damage in tumor cells. Antioxidant enzymes play a protective role by eliminating excessive ROS, thereby protecting tumor cells from oxidative damage. Therefore, targeting antioxidant enzymes are considered a potential strategy in current cancer therapeutics. PRDX1, an antioxidant enzyme, is overexpressed in several cancers and represents a promising target for the development of novel antitumor drugs. In this study, we identified five novel PRDX1 inhibitors (CPs 1–5) by integrated virtual screening strategy. Molecular docking techniques were used to dock these compounds from the database to the PRDX1 binding site, and CPs 1-5 were selected based on the docking scores. Among them, CP1 had the lowest docking score, indicating its strongest binding affinity to PRDX1. SAR analysis indicated that CPs 1-5 bind to PRDX1 through hydrogen bonding and hydrophobic interactions, with CP1 and CP2 showing more hydrogen bonding. In addition, MD simulations showed that CP1 had a more stable binding to PRDX1 than CP2. Meanwhile, CPs 1-5 exhibited significant PRDX1 inhibition with IC_50_ values in the nanomolar range, with CP1 having the most potent inhibitory activity. Selectivity assays showed that CP1 exhibited minimal inhibitory activity against other kinases, suggesting that CP1 selectively inhibits PRDX1. In particular, CP1 showed potent antiproliferative activity against A549, HepG2 and MCF-7 tumor cells without observable cytotoxicity in other normal cells, indicating its therapeutic potential. Meanwhile, no significant differences were observed in the levels of liver and kidney function indices in mice, suggesting that CP1 did not show obvious toxic and side effects. Celastrol is a well-studied natural product with antitumor activity that targets PRDX1. However, its further clinical application is severely limited by systemic toxicities, which are primarily due to off-target effects. Compared with celastrol, CP1 showed significantly stronger binding affinity to PRDX1 and more prominent antiproliferative activity against tumor cells. Meanwhile, CP1 selectively inhibits PRDX1 without causing off-target effects, thus exhibiting no apparent toxic responses. In summary, the strong correlation between these results provides compelling evidence for the reliability of our integrated virtual screening strategy in predicting lead compounds. Although this study successfully identified a novel PRDX1 inhibitor with potent antiproliferative effects *in vitro*, the lack of *in vivo* validation remains a critical limitation. Therefore, CP1 is a potential anticancer candidate that warrants a comprehensive evaluation of its *in vivo* therapeutic efficacy and safety profile in mouse models.

## 5 Conclusion

In this study, CPs 1-5 targeting PRDX1 were identified by structure-based virtual screening. These compounds inhibited PRDX1 at the nanomolar level, with CP1 being the most potent inhibitor and exhibiting high selectivity against PRDX1. The MST assay revealed that CP1 exhibited a significant binding affinity for PRDX1. The kinetic study showed that CP1 acted as noncompetitive PRDX1 inhibitor. MD simulations confirmed the binding stability of CP1 and PRDX1. MST assays revealed that CP1 had significant binding affinity for PRDX1. Notably, CP1 exhibited potent antiproliferative activity against tumor cells while showing no significant toxicity towards other normal cells. Meanwhile, CP1 showed no significant hepatotoxicity or renal toxicity in mice. In summary, we have identified a potential PRDX1 inhibitor that warrants further investigation for the treatment of cancer.

## Data Availability

The original contributions presented in the study are included in the article/Supplementary Material, further inquiries can be directed to the corresponding authors.
