# Supplementary material for: Discovery of novel and potent celastrol derivatives as PRDX1 inhibitors for cancer therapy through structure-based virtual screening
Source: Front Pharmacol. 2025 Jul 23;16:1625604. doi: 10.3389/fphar.2025.1625604 (PMC12325182; doi:10.3389/fphar.2025.1625604)
Supplement: Supplementary file 1 [file DataSheet1.docx]

**Supplementary Information**

**Discovery of novel and potent celastrol derivatives as PRDX1 inhibitors for cancer therapy through structure-based virtual screening**


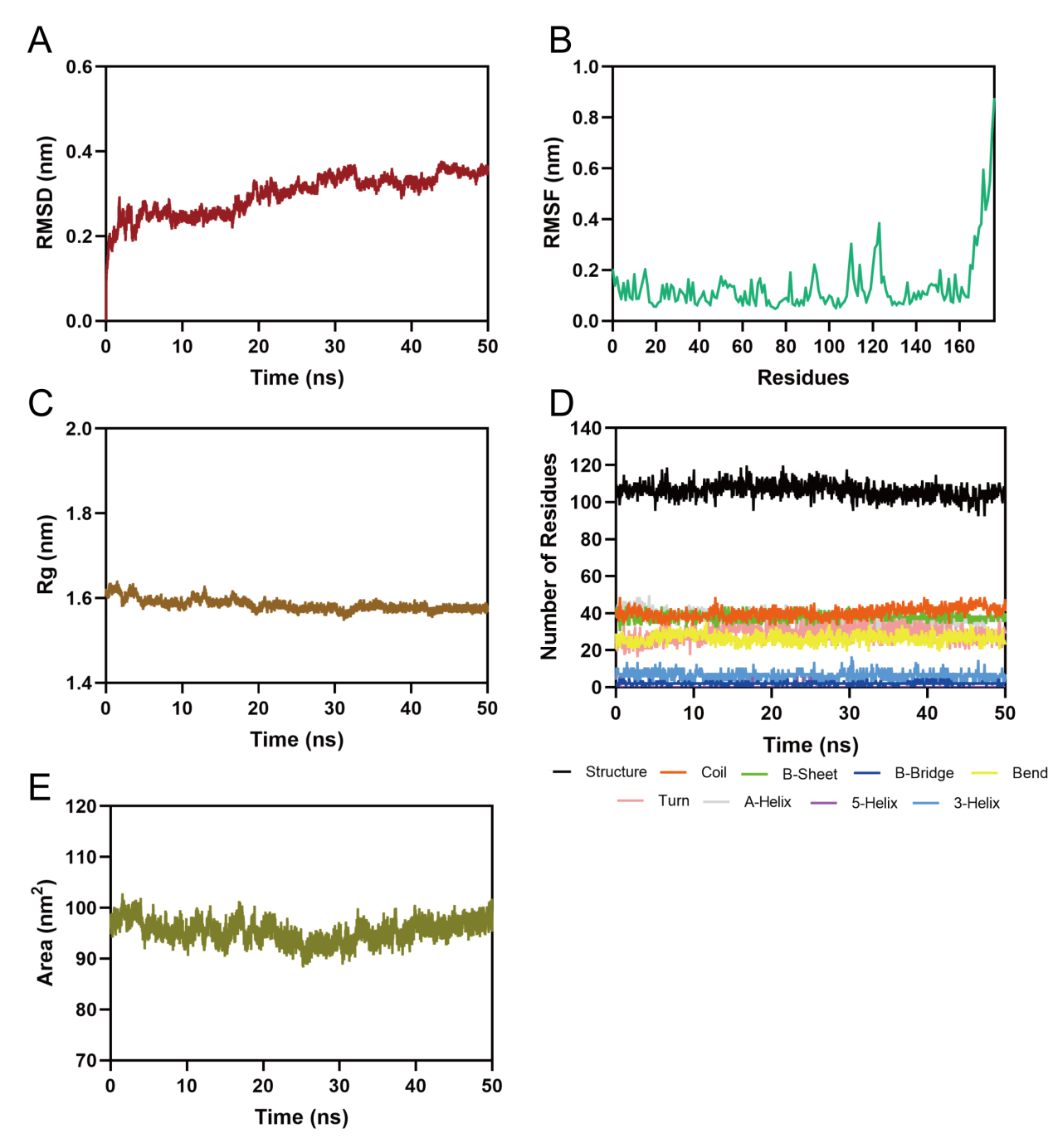


**FIGURE S1**. MD simulation of PRDX1 in complex with celastrol. (A) RMSD of the celastrol-PRDX1 complex; (B) RMSF of PRDX1 residues in the celastrol-PRDX1 complex; (C) Rg of PRDX1 in the celastrol-PRDX1 complex; (D) The secondary structures of PRDX1 in the celastrol-PRDX1 complex; (E) SASA of PRDX1 in the celastrol-PRDX1 complex.


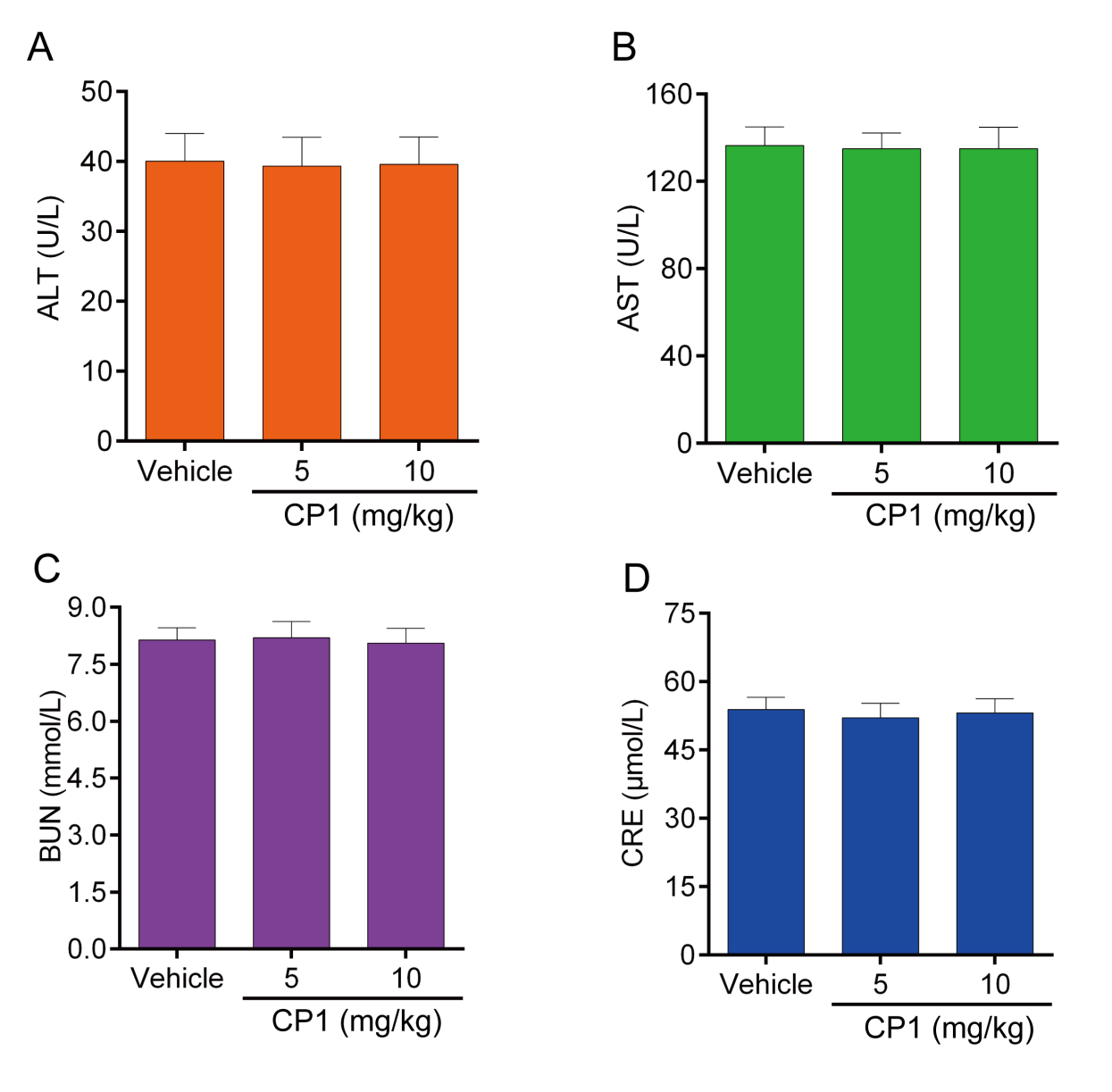


**FIGURE S2.** CP1 has no obvious adverse side effects on mice. (A-D) Biochemical parameters of mice in all groups. The results are represented as mean ± SD, n = 6.

**TABLE S1.** Selectivity profile of CP1 on other PRDX family members and a panel of kinases.

| **Target** | **CP1**  **IC_50_ (μM)** | **Target** | **CP1**  **IC_50_ (μM)** |
| --- | --- | --- | --- |
| PRDX2 | > 30 | EPHA8 | > 30 |
| PRDX3 | > 30 | EPHB1 | > 30 |
| PRDX4 | > 30 | EPHB2 | > 30 |
| PRDX5 | > 30 | EPHB3 | > 30 |
| PRDX6 | > 30 | EPHB4 | > 30 |
| ABL1 | > 30 | ERBB2 | > 30 |
| ABL2 | > 30 | ERBB4 | > 30 |
| ALK | > 30 | FER | > 30 |
| AXL | > 30 | FES | > 30 |
| BLK | > 30 | FGFR1 | > 30 |
| BMX | > 30 | FGFR2 | > 30 |
| BTK | > 30 | FGFR3 | > 30 |
| CSF1R | > 30 | FGFR4 | > 30 |
| CSK | > 30 | FGR | > 30 |
| DDR1 | > 30 | FLT1 | > 30 |
| DDR2 | > 30 | FLT3 | > 30 |
| EGFR | > 30 | FLT4 | > 30 |
| EPHA1 | > 30 | FRK | > 30 |
| EPHA2 | > 30 | FYN | > 30 |
| HCK | > 30 | NTRK3 | > 30 |
| IGF1R | > 30 | PDGFRA | > 30 |
| INSR | > 30 | PDGFRB | > 30 |
| INSRR | > 30 | PTK2 | > 30 |
| ITK | > 30 | PTK2B | > 30 |
| JAK1 | > 30 | PTK6 | > 30 |
| JAK2 | > 30 | RET | > 30 |
| JAK3 | > 30 | ROS1 | > 30 |
| KDR | > 30 | SRC | > 30 |
| KIT | > 30 | SRMS | > 30 |
| LCK | > 30 | SYK | > 30 |
| LTK | > 30 | TEC | > 30 |
| LYN | > 30 | TEK | > 30 |
| MERTK | > 30 | TNK2 | > 30 |
| MET | > 30 | TXK | > 30 |
| MST1R | > 30 | TYK2 | > 30 |
| MUSK | > 30 | TYRO3 | > 30 |
| NTRK1 | > 30 | YES1 | > 30 |
| NTRK2 | > 30 | ZAP70 | > 30 |

| **CP1** | **Concentration (nM)** | | | |
| --- | --- | --- | --- | --- |
|  | 0 | 0.6 | 1.5 | 3.8 |
| V_max_ (M/s) | 63.5 ± 2.73 | 56.2 ± 1.94 | 40.7 ± 2.42 | 26.8 ± 1.07 |
| K_m_ (μM) | 46.7 ± 1.87 | 60.1 ± 2.68 | 88.4 ± 3.77 | 122.6 ± 4.54 |
| α = 2.8 |  |  |  |  |

**TABLE S2.** The enzyme kinetic characteristics of CP1.

The data are presented as the mean ± SD, n = 3.

**TABLE S3.** Predicted free energies (kcal/mol) for the binding of celastrol to PRDX1.

| energy terms (kcal/mol) | celastrol |
| --- | --- |
| van der Waals energy | -23.85 ± 1.77 |
| electrostatic energy | -4.59 ± 2.50 |
| polar solvation energ | 13.93 ± 2.34 |
| nonpolar solvation energy | -2.62 ± 0.17 |
| total gas-phase free energy | -28.44 ± 3.06 |
| total solvation free energy | 11.31 ± 2.35 |
| total binding free energy | -17.12 ± 3.86 |

The data are presented as the mean ± SD.

**TABLE S4.** Determination of the binding affinities of CP1 and celastrol to PRDX1 by MST assays.

| **Compounds** | **PRDX1 (*K*_d_, nM)** |
| --- | --- |
| CP1 | 0.06 ± 0.001 |
| Celastrol | 0.32 ± 0.02 μM |

The data are presented as the mean ± SD, n = 3.

**TABLE S5.** Cytotoxic activity of CP1 and celastrol against tumor cells and normal cells incubated for 72 h by MTT assay.

| **Compounds** | **IC_50_ (μM)** | | | | |
| --- | --- | --- | --- | --- | --- |
|  | **HepG2** | **MCF-7** | **HEK293** | **LO2** | **HaCaT** |
| CP1 | 0.25 ± 0.02 | 0.27 ± 0.01 | > 100 | > 100 | > 100 |
| Celastrol | 1.28 ± 0.05 | 1.31 ± 0.06 | 5.76 ± 0.12 | 24.04 ± 0.69 | 17.38 ± 0.54 |

The data are presented as the mean ± SD, n = 3.
